# Bioconversion of 4-hydroxyestradiol by extradiol ring-cleavage dioxygenases from *Novosphingobium* sp. PP1Y

**DOI:** 10.1038/s41598-023-28908-2

**Published:** 2023-02-01

**Authors:** Francesca Mensitieri, Andrea Bosso, Fabrizio Dal Piaz, Bruno Charlier, Eugenio Notomista, Viviana Izzo, Valeria Cafaro

**Affiliations:** 1 Department of Medicine, Surgery and Dentistry “Scuola Medica Salernitana”, University of Salerno, Salerno, Italy; 2 Department of Biology, University Federico II of Naples, University Campus of Monte Sant’Angelo, Via Cinthia 4, Naples, Italy

**Keywords:** Biocatalysis, Environmental biotechnology, Environmental microbiology

## Abstract

Livestock breeding activities and pharmaceutical wastes lead to considerable accumulation of steroid hormones and estrogens in wastewaters. Here estrogens act as pro-cancerogenic agents and endocrine disruptors interfering with the sexual development of aquatic animals and having toxic effects in humans. Environmental bacteria play a vital role in estrogens degradation. Their wide reservoir of enzymes, such as ring cleavage dioxygenases (RCDs), can degrade the steroid nucleus, catalyzing the meta-cleavage of A, B or D steroid rings. In this work, 4 extra-diol ring cleavage dioxygenases (ERCDs), PP28735, PP26077, PP00124 and PP00193, were isolated from the marine sphingomonad *Novosphingobium* sp. PP1Y and characterized. Enzymes kinetic parameters were determined on different synthetic catecholic substrates. Then, the bioconversion of catechol estrogens was evaluated. PP00124 showed to be an efficient catalyst for the degradation of 4-hydroxyestradiol (4-OHE2), a carcinogenic hydroxylated derivate of E2. 4-OHE2 complete cleavage was obtained using PP00124 both in soluble form and in whole recombinant *E. coli* cells. LC–MS/MS analyses confirmed the generation of a semialdehyde product, through A-ring meta cleavage. To the best of our knowledge, PP00124 is the first characterized enzyme able to directly degrade 4-OHE2 via meta cleavage. Moreover, the complete 4-OHE2 biodegradation using recombinant whole cells highlighted advantages for bioremediation purposes.

## Introduction

**Figure 1:**
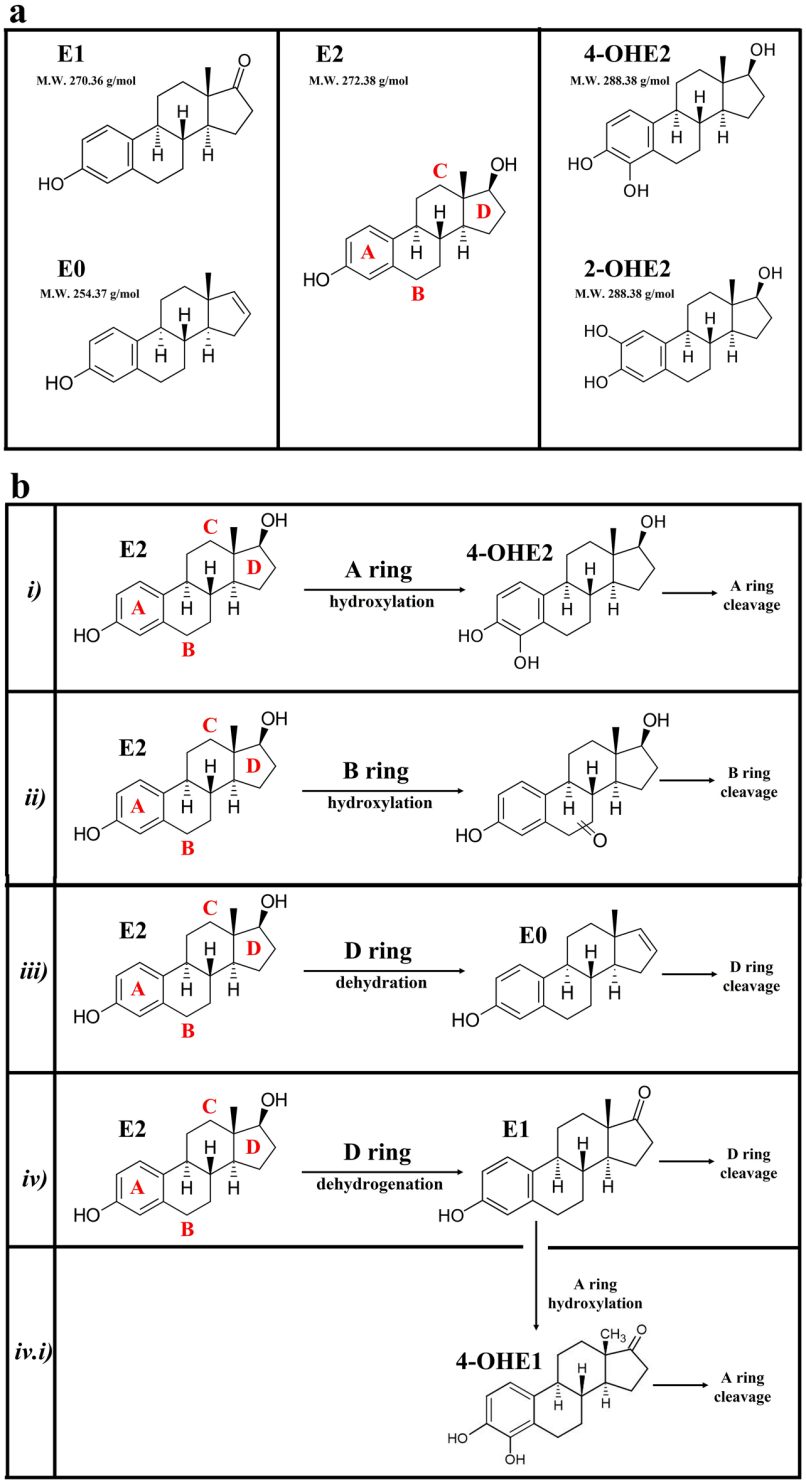
Main estrogenic compounds and E2 degradation pathways. (**a**) Structures and molecular weights (M.W.) of estrone (E1), 17 β-estradiol (E2), estratetraenol (E0), 4-hydroxyestradiol (4-OHE2) and 2-hydroxyestradiol (2-OHE2). Steroid rings A-D are indicated in red. (**b**) Major E2 degradation pathways. *i)* E2 A ring hydroxylation^19^. *ii)* E2 B ring hydroxylation^19^. *iii)* E2 D ring dehydration^20^. *iv)* E2 dehydrogenation to E1 and subsequent D ring dehydrogenation and cleavage^21^. *iv.i)* E2 dehydrogenation to E1 and subsequent A ring dehydrogenation and cleavage^18^. N.B. pathway *iv.i)* essentially follows the same A ring cleavage reaction as pathway *i)*. It is here separated since the cleavage substrate is 4-OHE1 in pathway *iv.i)* and 4-OHE2 in pathway *i)*.

Estrogens are the main steroid hormones responsible for the regulation of female reproductive system and the development of secondary sex characteristics^1^. In all vertebrates, estrone (E1), and 17β-estradiol (E2) are the main regulative hormones (Fig. 1a). E2, which has major estrogenic activity with plasma concentrations in female mammals up to 500 pg/mL, is among the main effectors for the reproductive system development in humans^2^. Estrogens are excreted in urine after hydroxylation or sulfonation^3^; several studies have shown that these compounds, and in particular their hydroxylated forms (4-hydroxyestradiol 4-OHE2 and 2-hydroxyestradiol 2-OHE2, Fig. 1a), may act as endocrine disruptors and carcinogens^4,5^.

The presence of these harmful compounds in wastewater and aquatic ecosystems is mostly due to farm animal excretions in livestock breeding activities^6^ and has recently become a major environmental concern^7–9^; these molecules seem in fact to be responsible for development, fertility, and reproductive function impairment in mammals, fish, and amphibian populations. Indeed, intersex fish populations have been observed worldwide in estrogen-contaminated ecosystems[^5^^,^^6^^,^^10–12^ and references therein].

The growing environmental concern has recently boosted the interest towards the biological processes involved in estrogens degradation, in particular of E2, and the identification of both new potential degraders and degrading enzymatic activities^13^. Degradation of estrogenic nuclei, through disruption of the aromatic A ring structure, is the key to hinder the interaction of these compounds with DNA, thus neutralizing their activity and consequent carcinogenic effect^13,14^. Microbial degradation processes play a vital role in determining the fate and transport of estrogens in the environment. Bacteria possessing the specific catalytic machinery for complete disruption of steroid nuclei in metabolic or co-metabolic mode play a central role for the conversion of estrogens to CO_2_^13–15^.

Estrogen biodegradation can be catalysed by different oxygenases and dehydrogenases, through different catabolic pathways. Ring cleavage dioxygenases (RCD) play a fundamental role in estrogen degradation, catalyzing the oxidative cleavage of hydroxylated estrogenic A rings. These microbial enzymes families play also a central role in the degradation pathways of mono- and polycyclic aromatic hydrocarbons and complex aromatic compounds (e.g. allowing the cleavage of environmental pollutants and lignin derivates in the environment). Oxygenases are usually enzymatic complexes that use cofactors and carry out redox reactions using oxygen as a co-reagent and NADH or iron as an electron donor^16^. The aromatic compounds degradation pathway in bacteria can be generally divided in two steps, indicated as upper and lower pathways^17^. The reactions of the upper pathways involve the activation of the aromatic ring by addition of one or two hydroxyl groups on two adjacent carbon atoms, often catalyzed by monooxygenases. Then, dioxygenases operate the oxidative ring cleavage of the catecholic compounds. Dioxygenases are commonly divided in intra- (IRCD) and extra-diol ring cleavage dioxygenases (ERCD)^18^. IRCD enzymes cleave the bond between the two hydroxyl groups generating a dicarboxylic compound (a derivative of *cis*-muconic acid). ERCDs catalyze the incorporation of O_2_ oxygen into catechol derivatives, thus cleaving one of the bonds adjacent to the diol and generating an alpha-hydroxy acid containing an aldehydic or ketonic group (a derivative of *cis*-muconic semialdehyde). These enzymes allow the conversion of dihydroxylated aromatic molecules into intermediates of the tricarboxylic acid cycle (TCA), which are readily metabolized by any type of organism. According to Yu and co-workers^14^, estrogens microbial degradation pathways are divided in four main routes, summarized in Fig. 1b. These degradative pathways could be also grouped depending on the steroid rings involved in the first degradative step: *i*) A ring: direct E2 hydroxylation in C4 to form 4-OHE2 and subsequent 4-OHE2 cleavage via the so-called 4,5-seco pathway, performed by ERCDs^19^; *ii*) B ring: E2 degradation starting from the hydroxylation of saturated B-ring and subsequent cleavage^19^; *iii*) D ring: direct E2 degradation via D-ring dehydration^20^; *iv*) D ring: in this pathway the first step always involves a dehydrogenation of E2 to E1. Subsequently E1 could be either dehydrogenated and cleaved on D ring (pathway *iv*)^21,22^ or hydroxylated to 4-OHE1 and cleaved on A ring through 4,5-seco pathway (pathway *iv.i*), similarly to what outlined in *i*) for 4-OHE2^19,23^.

In recent years, different works described the isolation of new microbial enzymes able to degrade E2, most of which are involved in the initial E2 dehydrogenation to E1, which is then hydroxylated and cleaved, as described in the *iv.i*) pathway^19,23–28^.

Bioprospecting activity is of utmost importance in shedding light on the biochemical routes involved in estrogen biodegradation and in expanding the enzymatic toolbox for biotechnological applications of these catalysts.

*Novosphingobium* sp. PP1Y, as other estrogen-degrader bacteria, is a marine alpha-proteobacterium member of the Sphingomonadales order, which was isolated from highly polluted waters in Pozzuoli (Italy). Several phenotypic traits of this strain, including the possibility to grow on complex mixtures of aromatic molecules dissolved in non-polar phases such as diesel and gasoline, suggest a unique metabolic versatility and the capacity to catalyze the biodegradation of complex polycyclic aromatic hydrocarbons (PAHs)^29^. *Novosphingobium* sp. PP1Y genome has been completely sequenced and annotated^30^, and its analysis reveal the presence of more than 40 putative *orfs* coding for mono- and dioxygenases, which represent an impressive reservoir of oxygenase activities likely responsible for the ability of this strain to degrade a wide range of mono-, bi-, tri-, tetra- and penta-cyclic aromatic compounds^30,31^.

In this work, the microorganism *Novosphigobium* sp. PP1Y was used as a novel source for the isolation of ERCDs active towards catechol estrogens. The genes coding for four ERCDs were recombinantly expressed in *E. coli;* the corresponding enzymes were characterized and the kinetic constants on different catecholic substrates were measured. Then, the bioconversion of catechol estrogens was evaluated either using cell extracts or whole recombinant cells. The ERCD PP00124 was identified and characterized as able to catalyze the complete bioconversion of 4-OHE2, both in soluble form and in whole recombinant cells, via meta cleavage.

## Results

### Four ERCDs from *Novosphingobium* sp. PP1Y, production, specific activity and kinetic parameters determination on catecholic substrates

The majority of known ERCDs belong to a heterogeneous protein family characterized by the presence of several subfamilies^32^. Each family has evolved to optimize the cleavage of a specific type of substituted catechol: 3- or 4-alkylcatechols, 3,4-dihydroxyphenylacetate, 2,3-dihydroxybiphenyl, 1,2-dihydroxynaphthalene, 3,4-dihydroxyphenanthrene.

Genome analysis of PP1Y strain^30^ revealed the presence of 7 *orf*s coding for putative ERCDs (three present in double copy with a 100% identity). A thorough phylogenetic study, supported by a homology modelling analysis, allowed to hypothesize the role of the seven enzymes in the metabolism of mono and polycyclic aromatic compounds^30^. In particular, it was hypothesized that *orfs* AT15671/AT31616, Mpl3065, AT15599/AT31688 and AT32663 code for, respectively, a putative (di)(methyl)catechol dioxygenase, a putative dihydroxybiphenyl dioxygenase and two dihydroxynaphtalene/dihydroxyphenanthrene dioxygenases. Herein, these enzymes will be named, respectively, PP00193, PP26077, PP00124 and PP28735.

The homology modelling/docking analysis described in D’Argenio et al.^30^ suggested that the active sites of the mentioned ERCDs should be able to host catechols with substituents at the positions 3 and/or 4, but the dimensions of the active site pocket progressively increase in the order PP00193 < PP26077 < PP00124 < PP28735. Very intriguingly, PP00124 and PP28735 showed active site pockets large enough to host dihydroxylated polycyclic aromatic hydrocarbons with 3–4 rings (e.g. phenanthrene, anthracene, benz[a]anthracene and chrysene) and 4-OHE2 that, from a steric point of view, is similar to 1,2-dihydroxychrysene (see Supplementary Figure S1). The determination of the specific activity of the four ERCDs on a limited panel of substrates, including 4-OHE2, confirmed the hypothesized roles of these proteins in the metabolism of Polycyclic Aromatic Hydrocarbon (PAH) in strain PP1Y^30^.

Therefore, the 4 ERCDs were selected as interesting activities to screen for estradiol bioconversion. Recombinant expression conditions were optimized in *E. coli* BL21(DE3) cells transformed with the corresponding pET22b( +) vectors, whose construction was previously described^30^. Expression conditions were optimized by testing different growth temperatures, induction times and IPTG concentrations, to obtain active enzymes. To select growing conditions, we evaluated the catalytic activity of ERCDs on 2,3-dihydroxybiphenyl (2,3-DHBP) as substrate in *E. coli* BL21(DE3) recombinant whole cells (Supplementary Figure S2 a), over the induction phase by IPTG. As showed in Supplementary Figure S2 b, all recombinant ERCDs were expressed in the soluble fraction, except for protein PP28735, which was mainly present in the insoluble fraction, as highlighted by SDS-PAGE analysis of the soluble and insoluble fractions after cell lysis (Supplementary Figure S2 b). The expression level of proteins in soluble form (Supplementary Table S1) was estimated to be 120, 40 and 100 mg/L of culture for PP26077, PP00124 and PP00193, respectively, but only 1–2 mg/L for enzyme PP28735.

ERCDs were then purified by an anionic exchange chromatography on a Q-Sepharose FF resin. A marked instability of all ERCDs in buffers without Fe(NH_4_)_2_(SO_4_)_2_ was observed, suggesting that, in the absence of exogenous iron in the buffer, the Fe(II) in the active site easily was lost or oxidized to Fe(III) thus leading to enzyme inactivation. A protocol for a quick “batch” chromatography was therefore set up, which involved the use of a buffer containing 30% glycerol and 0.1 mM Fe(NH_4_)_2_(SO_4_)_2_. As shown in Supplementary Figure S3 a, the yield of the purification procedures ranged from 44 to 95%, based on the evaluation of active enzyme purified (final total units) compared to the amount of active enzyme in cellular lysates (initial total units). SDS-PAGE analysis of purified proteins (Supplementary Figure S3 b) revealed the presence of contaminants up to 10–20% of total proteins for ERCDs PP26077, PP00124 and PP00193, whereas purified PP28735 showed a higher presence of contaminant proteins, most likely related to the low initial amount of protein in the soluble fractions of the induced cultures.

As all Ring Cleavage Dioxygenases are endowed with a Fe(II) ion as cofactor at the active site, and the lack of incorporation of Fe(II) into the mature protein, or its oxidation to Fe(III), causes total inactivation of the dioxygenase activity, we measured the iron content by Ferene S assay. Results reported in Supplementary Table S1 highlighted that total Fe(II) was found to be 60%, 100% and 88% for PP26077, PP00124 and PP00193 proteins, respectively. Recovery yield of protein PP28735 in the purified fractions was too low to measure the iron amount. Furthermore, iron assay allowed to estimate that 25% and 7% of PP26077 and PP00193 proteins, respectively, were iron-free, whereas the remaining fractions were endowed with Fe(III), leading to inactive enzymes. It is worth noting that purified PP00124 protein retained 100% Fe(II) at the end of expression and purification procedure, thus indicating a higher enzymatic stability compared to the other ERCDs.

Then, specific activity (S.A.) and kinetic parameters of purified ERCDs on the four catecholic substrates selected, 2,3-DHBP, 3-methylcatechol (3MC), 4-methylcatechol (4MC) and catechol (CAT) were determined (“Materials and Methods”). Specific activity of the purified RCDs was measured by monitoring the formation over time of the corresponding *cis*-muconic semialdehydes. Results shown in Table 1 suggested that proteins PP28735, PP26077 and PP00124 were mainly active on 2,3-DHBP and showed lower values of S.A. on the other monoaromatic substrates tested. Conversely, PP00193 was more active on CAT, 3-MC and 4-MC, as expected based on the modelling results^30^, which indicated that this enzyme has a smaller active site.

**Table 1:** Specific activity of purified ERCDs.

ERCD	S.A._2,3-DHBP_ mU/µg	S.A._3-MC_ mU/µg	S.A._4-MC_ mU/µg	S.A._CAT_ mU/µg
PP28735	36.13	1.75	2.73	0.66
PP26077	15.60	1.72	0.05	0.10
PP00124	32.70	13.54	4.48	7.12
PP00193	48.00	373.0	233.80	364.40

Specific Activity is here indicated as mU/µg. Errors were within 10% for all values reported. 2,3-dihydroxybiphenyl (2,3-DHBP), 3-methylcatechol (3-MC), 4-methylcatechol (4-MC) and catechol (CAT) were used as substrates.

Kinetic features of the 4 ERCDs, summarized in Table 2, showed that proteins PP00124 and PP00193 were endowed with the higher activity towards all substrates, with K_M_ values ranging from 30 to 1 µM. As expected, PP00193 seemed the best enzyme for the conversion of monoaromatic compounds and 2,3-DHBP, showing the higher *k*_*cat*_/K_M_ values for these substrates. Conversely, proteins PP28735 and PP26077 had a significant activity only on 2,3-DHBP, the larger substrate, while displaying K_M_ values higher than 450 µM on monoaromatic catechols. Indeed, these enzymes displayed *k*_*cat*_/K_M_ values for 2,3-DHBP conversion between 80 and 200 times lower compared to 3-MC. Protein PP00124 showed a comparable activity on all substrates tested, with a higher efficiency towards 2,3-DHBP.

**Table 2:** Kinetic constants of the ERCDs.

Substrates	Kinetic constants	ERCD			
		PP28735	PP26077	PP00124	PP00193
CAT	K_M_ (µM)	N.D	N.D	38.20	1.69
	*k*_*cat*_ (sec^−1^)	N.D	N.D	5.44	194.30
	*k*_*cat*_*/* K_M_ (sec^−1^ µM^−1^)	N.D	N.D	0.14	115
3-MC	K_M_ (µM)	451	2600	2.90	3.17
	*k*_*cat*_ (sec^−1^)	1.47	3	10.22	224.80
	*k*_*cat*_*/* K_M_ (sec^−1^ µM^−1^)	0.0033	0.0012	3.53	70.90
4-MC	K_M_ (µM)	N.D	N.D	1.80	1.89
	*k*_*cat*_ (sec^−1^)	N.D	N.D	2.97	120.00
	*k*_*cat*_*/* K_M_ (sec^−1^ µM^−1^)	N.D	N.D	1.65	63.50
2,3-DHBP	K_M_ (µM)	67.90	35.20	3.70	3.15
	*k*_*cat*_ (sec^−1^)	18.57	8.90	13.77	30.50
	*k*_*cat*_*/* K_M_ (sec^−1^ µM^−1^)	0.27	0.25	3.72	9.70

CAT (catechol), 3-MC (3-methylcatechol) 4-MC (4-methylcatechol) and 2,3-DHBP (2,3-dihydroxybiphenyl) were used as substrates. N.D., Not detectable. Errors were within 15% for each value reported.

### Bioconversion of catechol estrogens

Bioconversion of catechol estrogens using strain PP1Y ERCDs was tested. Substrates used for the bioconversions were 4-OHE2 and 2-OHE2 (Fig. 1a). These are derived from E2 hydroxylation, bearing the –OH substituents at positions 3, 4 and 2, 3 of the aromatic ring, respectively. Starting from the molecular docking analysis previously performed^30^, 4-OHE2 and 2-OHE2 should be better accommodated in PP28735 and PP00124 active sites. Therefore, these enzymes should display the higher affinity for the selected catechol estrogens. Preliminary data obtained in our previous work^30^ further supported this hypothesis. In this work, the S.A. of the four ERCDs was determined for 4-OHE (Table 5 in^30^). Data showed that PP28735 and PP00124 proteins were able to catalyse 4-OHE2 cleavage into its putative *cis-*muconic semialdehyde, whereas PP00193 and PP26077 proteins showed negligible activity.

**Figure 2:**
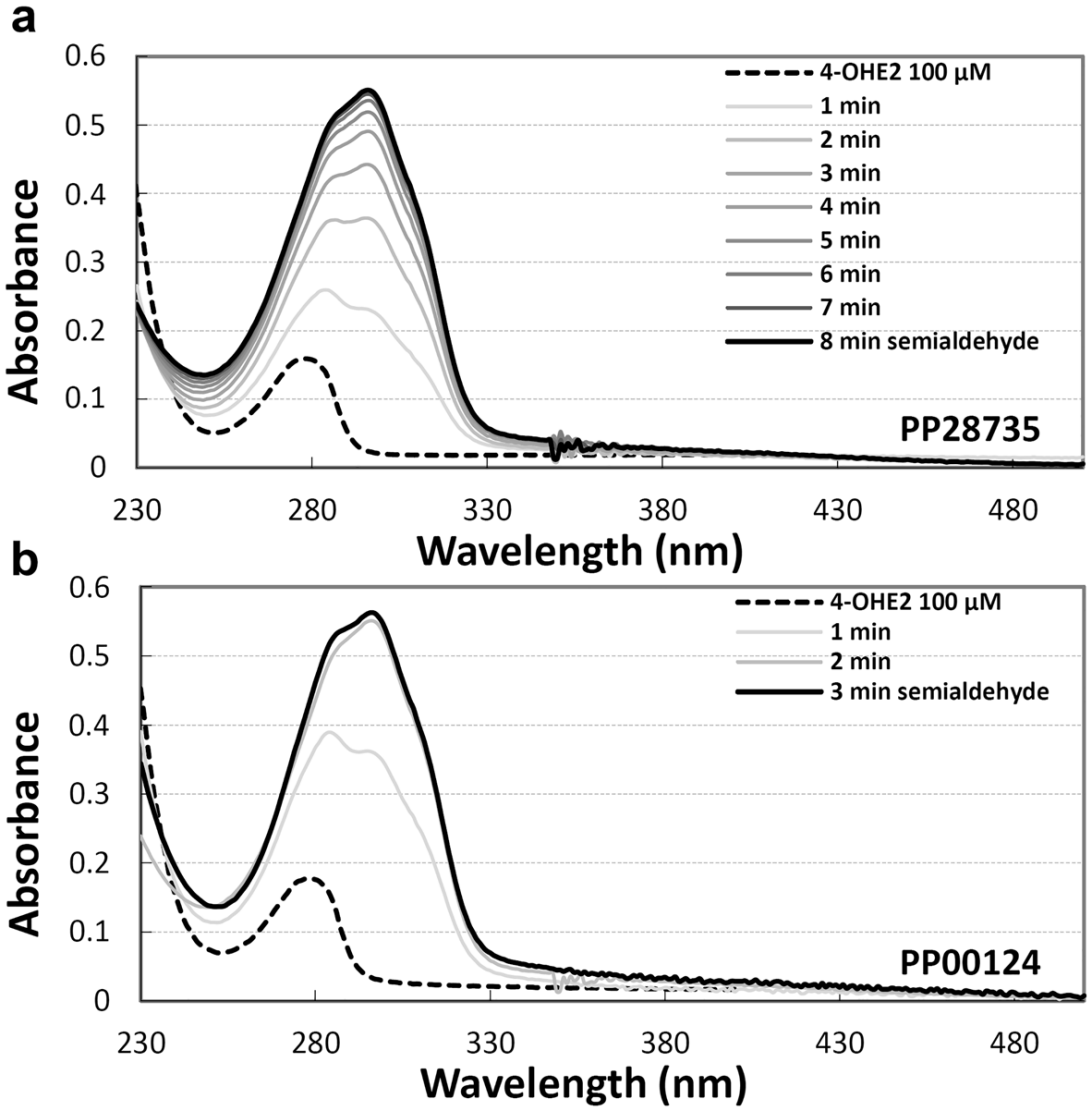
UV–vis spectra of the time-course of 4-OHE2 enzymatic conversion. Reactions were carried out using PP28735 (**a**) and PP00124 (**b**) enzymes in 1 mL of 50 mM Tris/HCl pH 7.5 buffer containing 100 μM 4-OHE2 (dashed black lines). The reactions were started by addition of purified enzymes. Semialdehyde production was monitored by the Scanning Kinetics program on Cary 100 UV–VIS spectrophotometer in a wavelength range from 230 to 500 nm, recording the absorption for 4–8 min, at 25 °C (gray scale lines). Bold black lines represent the spectra of semialdehyde at the end of reaction at pH 7.5 (λmax 298 nm).

To select the best enzyme for 4-OHE2 conversion, we performed time-course experiments (Fig. 2) carried out with PP28735 and PP00124 proteins by spectrophotometer analyses. Reactions, performed at pH 7.5 in the presence of 100 µM 4-OHE2, led to the formation of a reaction product endowed with λ_max_ at 298 nm (Fig. 2). Within 4 (PP00124) and 8 (PP28735) min incubation time, no further changes in spectra were observed, suggesting a complete conversion of 4-OHE2. Based on the spectral properties of the cleavage products, it could be hypothesized that the two enzymes catalyse the same reaction. However, it is worth noting that the semialdehydes obtained from meta cleavage of catechols appear, in general, as yellow products with λ_max_ between about 350 and 450 nm, whereas cleavage product from 4-OHE2 absorbed in spectra UV region with λ_max_ at 298 nm. It is well known that the yellow-coloured forms represent the dianionic form of semialdehydes, generally the most abundant at pH 7.5 at which the reactions were performed^33^. To verify the properties of the 4-OHE2 conversion products by PP28735 and PP00124 proteins, we alkalinized the reaction mixture by NaOH adding. The spectral properties in alkaline buffer reported in Supplementary Figure S4, highlighted products of yellow colour (λ_max_ at 417 nm) as expected for semialdehydes, thus confirming the ring cleavage reaction, and suggesting that a higher pH value is needed to obtain the dianionic yellow form.

We also performed kinetic analyses of 4-OHE2 conversion by the ERCDs to select the best catalyst. Product formation was monitored spectrophotometrically at 298 nm, wavelength at which no absorbance was recorded for 4-OHE2 substrate. As reported in Table 3, protein PP00124 showed the best kinetic parameters for the bioconversion of 4-OHE2 with sub-micromolar KM value, which prompted the set-up of a small-scale bioconversion protocol using cell lysates of *E. coli* expressing PP00124 as catalyst.

**Table 3:** Kinetic constants and specific activities of PP28735 and PP00124 on 4-OHE2.

Kinetic constants	PP28735	PP00124
K_M_ (µM)	1.90	0.75
*k*_*cat*_ (sec^−1^)	38.20	8.60
*k*_*cat*_*/* K_M_ (sec^−1^ µM^−1^)	20.10	11.43
*S.A. *(mU/µg)	42.50	12.85

Errors were within 15% for each value reported.

We also tested the ability of ERCDs to cleave 2-OHE2, but no conversion product was observed for any of the proteins, thus suggesting a selectivity of these enzymes toward 4-OHE2.

Based on the interesting kinetic properties of PP00124 protein, we further studied the kinetic of 4-OHE2 cleavage by PP00124 containing cell extracts performing HPLC analyses.

**Figure 3:**
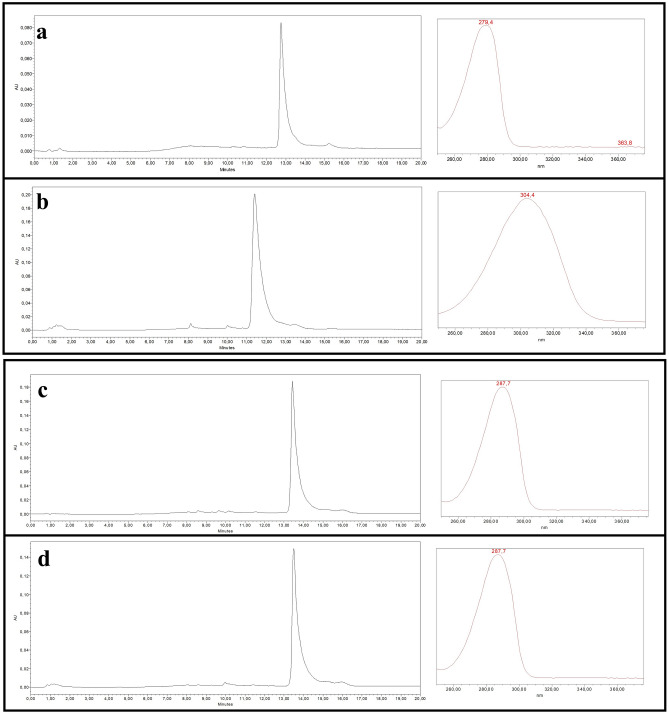
HPLC analysis of 4-OHE2 and 2-OHE2 bioconversion using recombinant PP00124 as catalyst. The assay was carried out in 50 mM Tris/HCl buffer pH 7.5 at 25 °C, with 100 µM and 200 µM of 4-OHE2 and 2-OHE2, respectively. Aliquots of the reaction mixtures were collected before the addition of the enzyme. Samples were acidified, diluted 100-fold, centrifuged, and analyzed in HPLC as negative controls (shown in panels (**a)** and (**c)** as blank reactions). Then, PP00124 cell lysate was added to start reactions. Product formation was followed spectrophotometrically over 15 min. Samples were then acidified, diluted 100-fold, centrifuged, and analysed by HPLC panels (**b)** and (**d**). (**a**) HPLC chromatogram of 4-OHE2 blank reaction and UV–vis spectrum of 4-OHE. (**b**) HPLC chromatogram of 4-OHE2 reaction: semialdehyde reaction product and corresponding UV–vis spectrum. (**c**) HPLC chromatogram of 2-OHE2 blank reaction and UV–vis spectrum. (**d**) HPLC chromatogram of 2-OHE2 reaction and corresponding UV–vis spectrum. All shown chromatograms were acquired at 280 nm.

The complete conversion of 4-OHE2 was verified spectrophotometrically. Bioconversion products obtained from the reaction were then analysed by HPLC. Results shown in Fig. 3 revealed that under our experimental conditions, the complete conversion of 4-OHE2 (retention time: 12.7 min and λ_max_: 279 nm, panel 3a) into a semialdehyde product (retention time: 11.4 min and λ_max_: 305 nm, panel 3b) was obtained, thus confirming the spectrophotometric analyses. On the contrary, when using 2-OHE2 as substrate (retention time: 13.4 min and λ_max_: 286 nm) (Fig. 3, panels c,d) no conversion was observed at any time of the reaction, in accordance again with spectrophotometric analyses. These data confirmed the tight specificity of PP00124 towards substrates bearing substituents in positions 3, 4 of the aromatic A rings.

**Figure 4:**
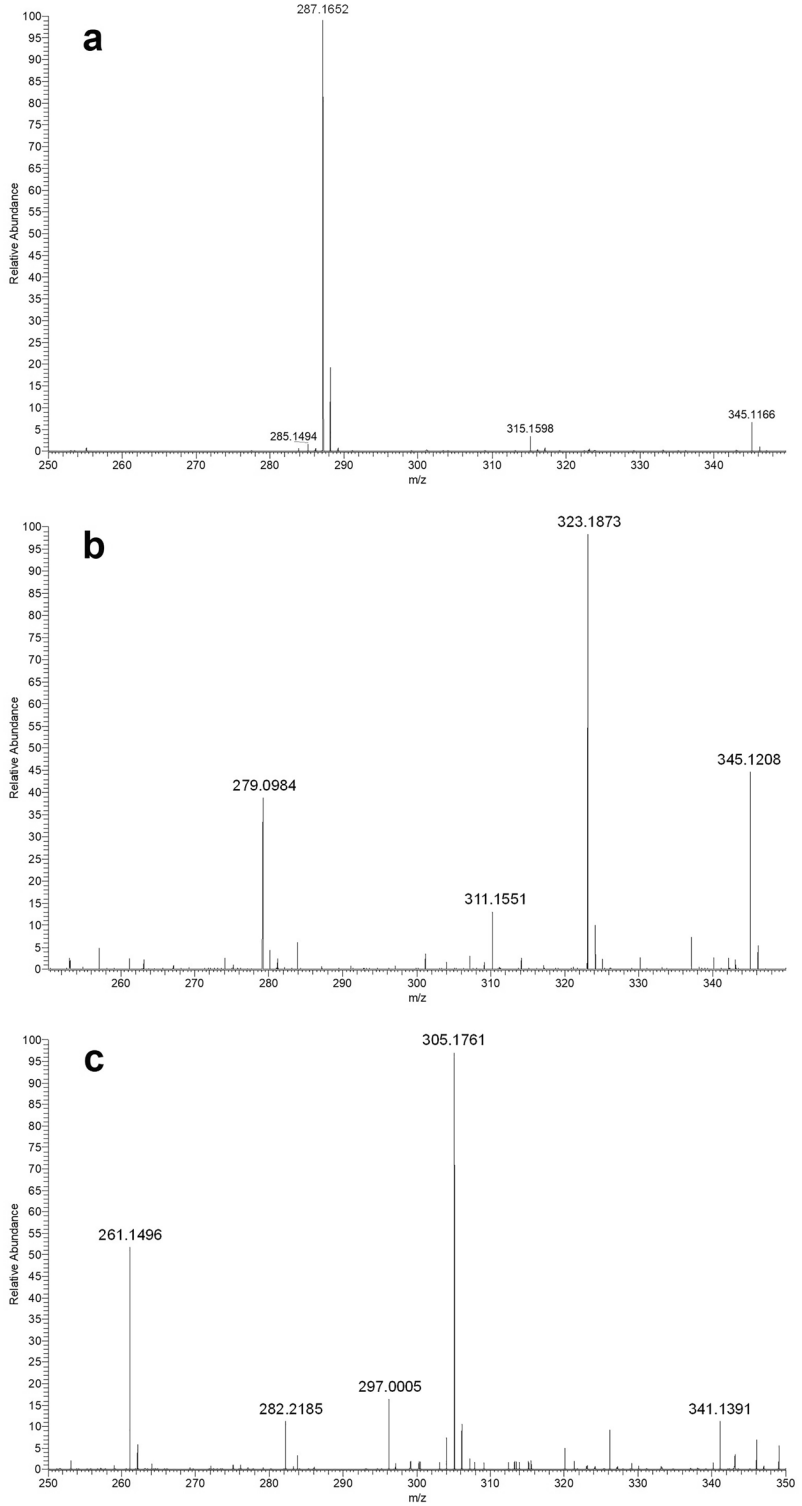
High resolution MS spectra of 4-OHE2 bioconversion products. Full mass (MS1) spectra were acquired in negative ion mode. (**a**) 4-OHE2 m/z spectrum (m/z 287.1652). (**b**) 4-OHE2 meta cleavage product m/z spectrum (m/z 323.1873). (**c)** 4-OHE2 cyclization product (hypothetical) m/z spectrum. (m/z 305.1761).

High resolution mass spectrometry analysis of conversion products was performed. Aliquots of the blank and end-point reactions were collected and subjected to a liquid–liquid extraction with ethyl acetate. The obtained extracts were analyzed by LC–MS/MS in negative ion mode. Two major reaction products generated by the PP00124-catalyzed bioconversion of 4-OHE2 were detected: two co-eluent species, with molecular weights of 306.1839 (measured m/z 305.1761, Fig. 4b) and 324.1951 (measured m/z 323.1873 , Fig. 4c), respectively.

**Figure 5:**
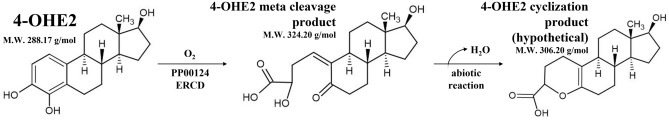
4-OHE2 hypothesized degradation mechanism using PP00124. The presence of the 4-OHE2 meta cleaved semialdehyde product (theoretical M.W. 324.1951), was verified by mass spectrometry. It is in line with ERCDs catalytic mechanism acting through an O_2_ insertion in the substrate. We hypothesize the generation of the second product identified, through a cyclization of the meta cleavage product, with the spontaneous nucleophilic attack of the hydroxyl on C4 onto the carbonylic group on C5. The hypothetic structure of this compound (theoretical M.W. 306.1839) is reported as 4-OHE2 cyclization product (hypothetical) .

These data confirmed the presence of the 4-OHE2 meta cleaved semialdehyde product (theoretical M.W. 324.1937), as expected based on the catalytic mechanism of ERCDs mediating the insertion of an O_2_ molecule in the substrate^34^. This is indicated in Fig. 4 as meta cleavage product. The second reaction product could be generated from the meta cleavage product, through a spontaneous nucleophilic attack of the hydroxyl on C4 onto the carbonylic group on C5, followed by dehydration (theoretical M.W. 306.1831). This is indicated in Fig. 4c as cyclization product (hypothetical). The hypothetic structures of these compounds and the proposed reaction are reported in Fig. 5.

### Bioconversion of 4-OHE2 using whole cells

Once the complete conversion of 100 μM 4-OHE2 using PP00124 was confirmed, we evaluated the possibility to obtain the 4-OHE2 bioconversion with whole cells as biocatalysts. To this purpose, *E. coli* recombinant cells expressing PP00124 were used.

**Figure 6:**
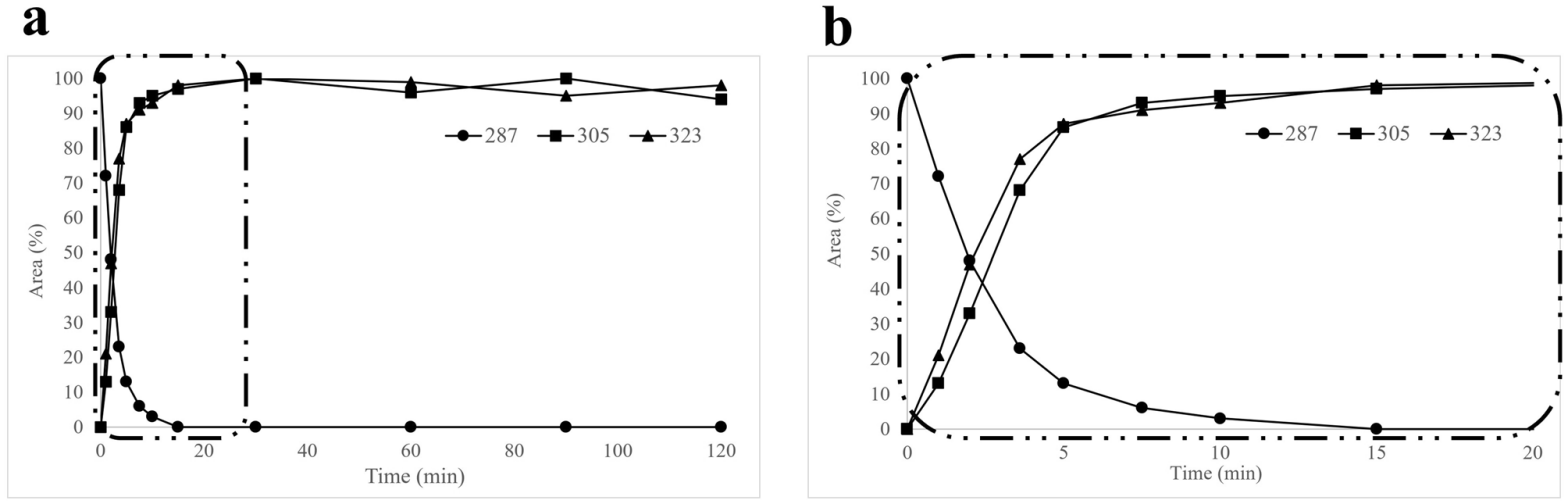
*E. coli* whole cells bioconversion of 4-OHE2 using recombinant PP00124 as catalyst. *E. coli* recombinant cells expressing P00124 were incubated at 37 °C with 100 μM 4-OHE2 in minimal medium. At different timepoints, aliquots of the supernatant were collected, and acidified. A liquid–liquid extraction with ethyl acetate was performed. HPLC–MS/MS quali-quantitative analyses was carried out. Full mass (MS1) spectra were acquired in high resolution negative ion mode. Peak areas of the extracted ion chromatograms for each compound and time point, were registered. Trend overtime of 4-OHE2 (m/z 287) and its cleavage products (m/z 305 and 323) are shown. (**a**) 2-h time-course bioconversion. (**b)** detail of 20 min bioconversion. Data are reported in Area (%) expressing relative abundance of the compounds. For each compound, the higher peak area registered was set as 100% area. Samples were analysed in triplicate and data were reported as the mean of the measured areas.

After recombinant expression, cells were assayed to test recombinant protein activity on 2,3-DHBP. A specific activity of 0.7 U_2,3DHBP_/OD was measured. 3.5 total Units_2,3-DHBP_ (5 OD_600_) of PP00124 expressing *E. coli* cells were incubated in minimal medium containing 100 μM (28 mg/L) 4-OHE2 and a time course experiment was carried out. The experiment was also performed with 10 OD_600_ cells of *E. coli* BL21(DE3) transformed with empty pET22b(+) plasmid as a negative control. Each time point of PP00124 cells and *E. coli* negative control reactions was extracted twice in ethyl acetate and analyzed by HPLC–MS/MS. No 4-OHE2 degradation was observed using *E. coli* cells transformed with the empty plasmid. Results obtained for PP00124 expressing cells are shown in Fig. 6. As observed in the reaction carried out using the purified enzyme, a fast decrease of 4-OHE2 (m/z 287) and formation over time of the two compounds generated by 4-OHE2 hydrolysis was evident (m/z 305 and 323). Interestingly, the kinetics accumulation of these two compounds were almost superimposable, thus suggesting that an equilibrium between these two species occurred.

The bioconversion rate performed using PP00124 expressing cells was significantly slower than the one carried out with the purified enzyme (outlined above). However, PP00124 k_cat_/K_M_ allowed 4-OHE2 complete conversion in 15 min using 3.5 Units_2,3-DHBP_ in 5 OD_600_. Noteworthy, to the best of our knowledge, no examples of 4-OHE2 whole cells biodegradation have been reported so far. Li and co-workers showed a complete degradation of E2 using a microbial consortium, after 3 days of incubation at 20 mg/L^35^.

## Discussion

ERCDs are receiving attention for their potential use in the bioconversion of estrogen and estrogen-like compounds. The identification and characterization of novel enzymatic activities is of utmost importance to shed light on the biochemical routes developed by bacterial cells to degrade these molecules, which could be harmful for human health. Moreover, ERCDs could be implemented also for biosynthetic applications, exploiting the site-specific ring cleavage performed by these enzymes^36,37^. Their substrate specificity might be fine-tuned for the biosynthesis of fine chemicals.

A previously published bioprospecting analysis of *Novosphingobium* sp. PP1Y genome revealed a remarkable abundance of RCDs probably allowing strain PP1Y to metabolize complex mixtures of catechols deriving from the simultaneous oxidation of several mono- and polycyclic-aromatic hydrocarbons, which are the preferred growth substrates for this strain^29,30^.

This work describes a kinetic characterization of four ERCDs previously isolated from *Novosphingobium* sp. PP1Y^30^. Their substrate specificity was investigated and complete 4-OHE2 biodegradation was obtained using protein PP00124, with either recombinant whole cells or cell lysates.

Kinetic parameters on CAT, 3-MC, 4-MC and 2,3-DHBP of the 4 ERCDs were determined. Data obtained confirmed the homology modelling/docking analysis previously performed for the enzymes in D’Argenio et al. (^30^ and Supplementary Figure S7 therein). Indeed, protein PP00193 had the higher efficiency for the degradation of monoaromatic compounds, showing *k*_*cat*_/K_M_ values ranging from 115 to 64 s^−1^ µM^−1^. These values appeared to be comparable or even higher than those described for similar enzymes from *Pseudomonas putida* mt-2 and *Burkholderia* sp. LB400^38–40^. Protein PP00124 showed a comparable activity on all substrates we have tested, but with a higher efficiency towards 2,3-DHBP. Overall, even though the *k*_*cat*_/K_M_ values of proteins PP00124 and PP00193 towards this substrate were not higher than the ones described for other biphenyl dioxygenases (as those from *Sphingomonas* sp. RW1 and *Burkholderia* sp. LB400), these proteins from strain PP1Y are endowed with the ability to cleave several 3- and/or 4-substituted catechols, which has not been described for other enzymes already characterized in literature^40,41^. Conversely, protein PP26077 had a very low activity on these substrates and as expected, it seems to have a higher affinity for molecules with one substituent perpendicular to the aromatic ring, as in 2,3-DHBP.

For what concerns 4-OHE2 cleavage, PP00124 resulted to be the best catalyst. HPLC–MS/MS analysis highlighted the complete substrate bioconversion after 15 min reaction. No conversion of 2-OHE2 was observed, thus suggesting a tight specificity of the enzyme for 2,3 substituted A rings structures. The proximal estradiol cleavage of 3- or 4- substituted catecholic ring to form 2-hydroxymuconate semialdehyde with the insertion of two atoms of dioxygen was observed, as expected for the ERCDs reaction mechanism, by the generation of a 323.187 m/z product (287 + 36 = 323 m/z ion)^34,38^. Results showed however, the generation also of a 305.176 m/z compound, probably derived by spontaneous intramolecular cyclization, with elimination of one water molecule. In Fig. 5, a representation of the hypothesized structure of the cyclization product is shown. The two products appeared to be generated at the same time and to remain in equilibrium over time during the reaction (Fig. 6). The hypothesis could be advanced that the cyclization product is generated from the semialdehydic meta-cleavage product through a nucleophilic attack and dehydration and the formation of a heterocyclic ring in A position (Fig. 5). Other works described the formation of a similar heterocyclic compound, pyridinestrone acid, through a spontaneous abiotic reaction in presence of a nitrogen donor in the medium^24^. Therefore, the generation of a similar secondary product such cyclization product is in line with data previously reported. Even though further analyses would be needed for the complete characterization of the reaction mechanism, our data suggested PP00124 from *Novosphingobium* sp. PP1Y to be an efficient biocatalyst for 4-OHE2 biodegradation. To the best of our knowledge, here we describe the isolation and identification in *Novosphingobium* sp. PP1Y of the first ERCD able to directly degrade 4-OHE2 via meta cleavage and probably involved in the *i*) 4,5 seco pathway. Even though the biodegradation of 4-OHE2 was previously reported and identified as a possible natural degradation pathway in *Rhodococcus* sp. ED6 and *Sphingomonas* sp. ED8 by Kurisu et al.^19^, to the best of our knowledge PP00124 from *Novosphingobium* sp. PP1Y is the first characterized ERCD cleaving this substrate. Conversely, several recent works describe the characterization of microbial enzymes involved in the *ii*), *iv*) and *iv.i*) degradation pathways (Fig. 1) involving the cleavage of the estrogenic nucleus of 4-OHE1. However, in these examples an initial D ring dehydrogenation on E2 and conversion in E1 is always the first step (Fig. 1)^24,26–28^.

Finally, in this work whole cells bioconversion experiments highlighted some interesting advantages in using recombinant whole cells, over native bacteria, as biocatalysts. Several works reported estrogen biodegradation using different environmental strains or consortia^35^. Even though this approach could be highly beneficial in bioremediation purposes, here we highlight the advantages of using recombinant strains to improve the biodegradation of specific compounds. In fact, *E. coli* whole cells in which the recombinant expression of PP00124 was induced were able to effectively degrade 4-OHE2 with high efficiency and in short time, thus suggesting that the whole cells bioconversion protocol might be foreseen as an efficient approach to avoid the time and cost consuming steps of protein purification. Moreover, a similar approach could be hypothesized for the degradation of different recalcitrant compounds which are still poorly metabolized from wild-type environmental strains and consortia.

## Materials and methods

### Generals

Bacterial cultures, plasmid purifications and transformations were performed according to Sambrook et al.^42^. Protein concentration was measured with the Bio-Rad Protein Assay using bovine serum albumin (BSA) as a standard. Denaturing polyacrylamide gel electrophoresis (SDS-PAGE) was carried out using standard techniques and stained by Coomassie brilliant blue G-250. 4-OHE2 and 2-OHE2 standards were purchased from Cayman Chemicals. Bacterial growth was followed by measuring optical density at 600 nm, OD/mL, referred to as OD_600_. LB rich medium (Luria Bertani medium) was prepared as described in Sambrook et al.^42^.

### Recombinant expression and purification procedures

Plasmid pET22b(+) containing the *orfs* coding for proteins PP28735, PP26077, PP00124 and PP00193 were used to transform *E. coli* BL21(DE3) competent cells according to Sambrook et al.^42^. Colonies were inoculated in a sterile 50 mL Falcon tube containing 12.5 mL of LB medium supplemented with 100 µg/mL ampicillin (amp). Cultures were incubated at 37 °C in constant shaking up to ~ 0.7 OD_600_. The preinoculum was diluted 1:50 in 500 mL of LB/ amp and incubated with constant shaking at 37 °C up to OD_600_ of 0.6–0.7. Expression was induced by adding 0.1 mM Fe(SO_4_)_2_(NH_4_)_2_ and IPTG between 0.025 and 0.4 mM. Growth was continued at 28 °C or 37 °C for further 2 or 4 h (Supplementary Figure S2). Cells were then collected by centrifugation (5524 × g for 15 min at 4 °C) and cell pellets were stored at − 80 °C until needed. Cell paste obtained from large scale expression experiments were first suspended in lysis buffer (50 mM Tris/HCl, pH 7.5, 10% ethanol, 30% glycerol, 5 mM DTT and 0.1 mM Fe(SO_4_)_2_(NH_4_)_2_) at a final concentration of 50–100 OD_600_, and then disrupted by sonication (20 times for a 15″ on and 55″ off cycle, on ice). Soluble and insoluble fractions of induced cultures were separated by centrifugation at 22,100 × g for 30 min at 4 °C and the supernatant was collected and filtered through a 0.45 μm PVDF Millipore membrane.

PP26077, PP00124 and PP00193 proteins were batch purified on Q-Sepharose FF anionic exchange resin (Pharmacia Biotech). Samples were incubated 1 h with the resin under constant shaking at 4 °C, and the unbound proteins were removed by centrifugation and three subsequent washes in lysis buffer. Elution was performed by stepwise method in lysis buffer containing 0.2, 0.4, 0.6 and 0.8 M NaCl. The resin was incubated 3 times with each solution for 10 min under constant shaking at 4 °C. Fractions were collected by centrifugation and stored at − 80 °C under nitrogen atmosphere. Purity of samples were assessed by SDS-PAGE. The presence of active recombinant ERCDs was detected by performing the enzymatic assay on 2,3-DHBP, as below described. Furthermore, all manipulations were performed under a constant nitrogen flow to prevent excessive iron oxidation.

Protein PP28735 was purified by a Q Sepharose FF column chromatography. Protein elution was carried out by a linear gradient from 0 to 0.5 M NaCl in lysis buffer at a flow rate of 15 mL/h. The presence of the active recombinant ERCD was detected by performing the enzymatic assay on 2,3-DHBP. Relevant fractions were analysed by SDS-PAGE, pooled, purged with nitrogen, and stored at − 80 °C until use.

Total iron content was determined colorimetrically by complexation with Ferene S^43^ (3-(2-pyridyl) -5,6-bis [2-(5-furyl sulphonic acid)] -1,2,4-triazine, disodium salt), a specific chelating agent for Fe (II), which gives rise to the formation of a colored complex whose absorption is measured at a wavelength of 593 nm (ε_593nm_ = 34.32 mM^−1^ cm^−1^). To determine the total amount of Fe [Fe (II) + Fe (III)], the assay was carried out also in the presence of Vitamin C (final concentration 6 mM) to reduce Fe (III) to Fe (II). The amount of Fe (III) was calculated by subtracting the amount of Fe (II) (measured in the absence of Vitamin C) from the total amount of Fe (measured in the presence of Vitamin C). Calibration curves for Fe (II) and Fe (III) were obtained by testing known quantities (5–20 nmol) of Fe(NH_4_)_2_(SO_4_)_2_ and FeCl_3_, respectively. Since the chromatographic step required the presence of Fe (II) and DTT in the elution buffer, it was necessary to remove these reagents. To this purpose, low pressure molecular exclusion chromatography was performed on a PD-10 pre-packed column from Pharmacia (column size: 0.8 cm × 5 cm with a total volume of resin equal to 9.1 mL). The elution was carried out at room temperature in 50 mM pH 7.5 Tris/HCl buffer, 10% Glycerol, 0.2 M NaCl.

### Enzyme activity assays

#### Activity assays of recombinant ERCDs using four different catecholic substrates

Activity of ERCDs was determined at 25 °C by measuring their specific activity (S.A.) using CAT, 3-MC, 4-MC and 2,3-DHBP. Substrates stock solutions were prepared 100 mM in water for CAT, 3MC, 4MC, and in dimethylformaldehyde (DMF) for 2,3-DHBP. Their concentration was measured spectrophotometrically in 10 mM HCl. Samples were assayed at 25 °C in a total volume of 1 mL of 50 mM Tris/HCl pH 7.5 containing substrate at a final concentration of 1 mM. Reactions were started by adding variable amounts of whole recombinant cells (0.03 to 0.3 OD_600_), soluble fractions from cell lysates (0.03 to 0.3 OD_600_) or purified proteins (1–50 µg) and monitored spectrophotometrically over time for 3 min following the formation of the corresponding *cis*-muconic semialdehydes products. Absorbance was recorded at the λ_max_ of each substrate product (Supplementary Table S2) every 5 s. The λ_max_ and the extinction coefficients (ε) used to calculate the amount of each product obtained were reported in Supplementary Table S2. One unit of enzyme was defined as the amount of the enzyme that releases one µmole of *cis*-muconic semialdehyde per min at 25 °C.

#### Activity assays of recombinant ERCDs using 4-OHE2 and 2-OHE2

Substrates stock solutions were prepared 100 mM in ethanol. Their concentration was spectrophotometrically measured in 10 mM HCl by recording absorbance at their λ_max_ corresponding to 276 nm and 286 nm for 4-OHE2 and 2-OHE2, respectively. ε of 3-MC and 4-MC (Supplementary Table S2) were used for 4-OHE2 and 2-OHE2, respectively. Activity assays were carried out in 50 mM Tris/HCl buffer pH 7.5, at 25 °C, with variable amounts of ERCDs-containing whole recombinant cells (0.03 to 0.3 OD_600_), soluble fractions from cell lysates or purified proteins (1–50 µg, 30–400 mU_2,3-DHBP_), in the presence of 100 µM and 200 µM of 4-OHE2 and 2-OHE2, respectively, that represent their solubility limits in aqueous buffers.

Time-course experiments of 4-OHE2 (100 µM) and 2-OHE2 (200 µM) conversion were carried out on Varian Cary 100 UV–Vis spectrophotometer. Reactions were started by adding the purified ERCDs (1–50 µg) and the formation of the products was monitored at pH 7.5 by the Scanning Kinetics program in a wavelength range from 230 to 500 nm. The 4-OHE2 (λ_max_ 285 nm) conversion to semialdehyde (λ_max_ 298 nm) was followed by recording the absorption spectra for 4–8 min, at 25 °C. At the end of 4OHE2 cleavage, to convert the semialdehyde into the yellow dianionic form (λ_max_ 417 nm, at alkaline pH), NaOH (100 mM final concentration) was added, and the spectrum was recorded.

Furthermore, to simplify the identification of 4-OHE2 substrate and its product by HPLC analyses, the reaction containing the semialdehyde obtained after 10 min incubation as above described, was added by 1% formic acid and the spectrum was recorded. λ_max_ values of 280 and 305 nm were found for 4-OHE2 and its semialdehyde, respectively at acidic pH.

To allow the specific activity and kinetic constants determination and to quantify the semialdehyde production by ERCDs at pH 7.5 (over the reaction incubation time) and at pH 12 (at the end of reaction), the ε for 4-OHE2 semialdehydic product at pH 7.5 and pH 12 were experimentally determined. In brief, total conversion of different estradiol substrate concentrations (20 µM, 40 µM, 60 µM, 80 µM and 100 µM) was carried out using 400 mU_2,3DHBP_ of ERCD PP00124 in 50 mM Tris/HCl, pH 7.5 buffer. Spectra were recorded by Scanning Kinetics program as above described and extinction coefficients were determined (ε_298 nm_ at pH 7.5 = 9,1 mM^−1^ cm^−1^; ε_417 nm_ at pH 12 = 21,78 mM^−1^ cm^−1^) at the maximum wavelengths. One unit of enzyme activity was defined as the amount of the enzyme that releases one µmole of semialdehyde per min at 25 °C.

#### Kinetic parameters determination of recombinant ERCDs

Kinetic parameters were obtained in 50 mM Tris/HCl pH 7.5 buffer using between 1 and 50 µg of purified proteins and CAT, 3-MC, 4-MC and 2,3-DHBP as substrates in the range 0.5 µM–16 mM. 4-OHE2 was used at final concentrations ranging from 0.5 to 100 µM (solubility limit in water). Reactions were monitored for 3 min at 25 °C at 375 nm (CAT; ε_375nm_ = 33 mM^−1^ cm^−1^), 388 nm (3-MC; ε_388nm_ = 13.8 mM^−1^ cm^−1^), 382 nm (4-MC; ε_382nm_ = 28.1 mM^−1^ cm^−1^), 434 nm (2,3-DHBP; ε_434nm_ = 13.2 mM^−1^ cm^−1^) and 298 nm (4-OHE2; ε_298nm_ = 9.1 mM^−1^ cm^−1^). Extinction coefficients used were listed in Supplementary Table S2. All kinetic parameters were determined by a non-linear regression curve using GraphPad Prism 7.

### HPLC analysis of 4-OHE2 and 2-OHE2 conversion by PP00124

Enzymatic conversion of 4-OHE2 and 2-OHE2 was performed using PP00124 enzyme. Reactions were carried as outlined in the previous paragraph. Briefly, the assay was carried out in 1 mL of 50 mM Tris/HCl buffer pH 7.5 at 25 °C, in the presence of 100 µM and 200 µM of 4-OHE2 and 2-OHE2, respectively. An aliquot (0.5 mL) of the reaction mixtures was collected before the addition of the enzyme, and 1% final concentration of formic acid was added. Samples were diluted 100-fold, centrifuged at 22,100 × g for 5 min and run in HPLC as a negative control (blank reactions). Then, PP00124 cell lysate were added to the samples to initiate reactions. Product formation was followed spectrophotometrically over a total time of 15 min, between 230 and 500 nm, recording spectra every 5 s. Afterwards, reactions were stopped by adding 1% formic acid. Semialdehydes spectra were recorded at acidic pH and the samples were diluted 100-fold, centrifuged at 22,100 × g for 5 min and analysed by HPLC.

A Waters 1525 binary pump HPLC with a photodiode array detector (Waters 2996) equipped with a C18 ultrasphere column (Beckman Coulter 4.6 mm × 25 cm) was used. Two hundred microliters of each sample were injected. A linear gradient elution was performed at a flow rate of 1 mL/min, using a mobile phase composed of 0.1% formic acid in water (solvent A) and 0.1% formic acid in methanol (solvent B). Elution of 4-OHE2, 2-OHE2 and corresponding products was carried out using the following gradient: isocratic elution at 10% solvent B for 3 min, from 10 to 50% solvent B in 3 min, from 50 to 75% solvent B in 15 min, from 75 to 90% solvent B in 1 min, isocratic elution at 90% solvent B for 1 min, from 90 to 10% solvent B in 1 min, isocratic elution at 10% solvent B for 3 min. Spectra of the eluting solvent were registered with the photodiode array detector between 230 and 400 nm. Chromatograms were generated monitoring absorbance at 280 nm, since both substrates and product display absorption at that wavelength.

Finally, aliquots of the blank and endpoint reaction mixtures were also collected for LC–MS/MS analysis. Aliquots were centrifuged at 22,100 × g for 30 s. Supernatants were collected and formic acid was added at a final 1% concentration to stop the reaction; samples were then stored at − 20 °C overnight. Then a liquid–liquid extraction with ethyl acetate was performed to purify 4-OHE2 and the reaction products. Five volumes of ethyl acetate were added to the samples and vortexed for 20 s. Samples were centrifuged at 22,100 × g for 10 min and the organic phase was collected. The aqueous phase was then subjected to a second liquid–liquid extraction with the same protocol. All organic phases were vacuum-dried and suspended in 60 μL of 50% MeOH for LC–MS/MS analysis.

### *E. coli*—PP00124 whole cells bioconversions of 4-OHE2

Recombinant expression of protein PP00124 was induced as previously described. Two hundred and fifty millilitre cultures of *E. coli* BL21(DE3) transformed with pET22b( +) empty plasmid, were also grown as a negative control. After 2 h of induction, cells were collected by centrifugation and suspended at 5 OD_600_ in minimal medium (50 mM potassium phosphate, pH 7.0) supplemented with 0.4% glucose and 1 mM Fe(NH_4_)_2_(SO_4_)_2_. Finally, 100 μM 4-OHE2 was added to the medium and cells were incubated at 37 °C under constant shaking. Aliquots of 500 μL were collected at the following timepoints: 0, 1, 2, 3.5, 5, 7.5, 10 15-, 30-, 60-, and 120-min. Aliquots were centrifuged at 22,100 × g for 30 s. Supernatants were collected and formic acid was added at a final 1% concentration to stop the reaction; samples were then stored at − 20 °C overnight. Then a liquid–liquid extraction with ethyl acetate was performed to purify 4-OHE2 and the reaction products obtained, following the same procedure outlined in the previous paragraph; Five volumes of ethyl acetate were added to the samples and vortexed for 20 s. Samples were centrifuged at 22,100 × g for 10 min and the organic phase was collected. The aqueous phase was then subjected to a second liquid–liquid extraction with the same protocol. All organic phases were vacuum-dried and suspended in 60 μL of 50% MeOH for LC–MS/MS analysis.

### LC–MS/MS analyses

LC–MS/MS quali-quantitative analyses of the reaction mixtures from different bioconversion experiments were performed on a LTQ-Orbitrap ESI mass spectrometer (Thermo-Fisher Scientific) coupled with an Accela UHPLC system (Thermo-Fisher Scientific). Chromatographic separation was carried out on a Kinetex C18 column (150 × 2 mm, 2.6 μm) (Phenomenex) using aqueous 0.1% formic acid (A) and methanol (B) as mobile phases, a linear gradient from 5 to 30% of B in 10 min and a 200 μL/min flow rate. Full mass (MS1) spectra were acquired in high resolution negative ion mode, over the m/z range from 125 to 500. MS/MS spectra were acquired for the most intense ion detected in the MS1 spectra (dependent scan mode), by in-trap fragmentation.

Relative abundance of 4-OHE2 and reaction products was estimated by generating an extracted ion chromatogram for each compound (m/z 287.2 for 4-OHE2, for 323.2 for 4-OHE2 meta cleavage product and 305.2 for 4-OHE2 cyclization product (hypothetical).

For each compound, the highest peak area registered was assumed as 100% area. Area % at the other time points were calculated compared to the highest peak area. Relative abundances obtained at the different time points were plotted in function of time.

Samples were analysed in triplicate and data were reported as the mean of the measured areas.

## Supplementary Information

Supplementary Information.

## Data Availability

ERCDs protein sequences are available on UniProt database. The Accession Numbers are as follow: F6IHX1, F6ID78, F6IHP2 and F6IHN4 for PP28735, PP26077, PP00124 and PP00193 proteins, respectively.
