# Supplementary material for: Bioconversion of 4-hydroxyestradiol by extradiol ring-cleavage dioxygenases from Novosphingobium sp. PP1Y
Source: Sci Rep. 2023 Feb 1;13:1835. doi: 10.1038/s41598-023-28908-2 (PMC9892492; doi:10.1038/s41598-023-28908-2)
Supplement: Supplementary file 1 — Supplementary Information. [file 41598_2023_28908_MOESM1_ESM.pdf]

Supplementary information

Bioconversion of 4-hydroxyestradiol by extradiol ring-cleavage dioxygenases from *Novosphingobium* sp. PP1Y

Francesca Mensitieri[1], Andrea Bosso[2], Fabrizio Dal Piaz[1], Bruno Charlier[1], Eugenio Notomista[2], Viviana Izzo[1], Valeria Cafaro [2*].

[1] Department of Medicine, Surgery and Dentistry “Scuola Medica Salernitana”, University of Salerno, Italy.
[2] Department of Biology, University Federico II of Naples, Italy.

[*]Corresponding author:

Prof. Valeria Cafaro, PhD
Department of Biology
University Campus of Monte Sant’Angelo
Via Cinthia 4
Naples, Italy.
vcafaro@unina.it

Supplementary Figures

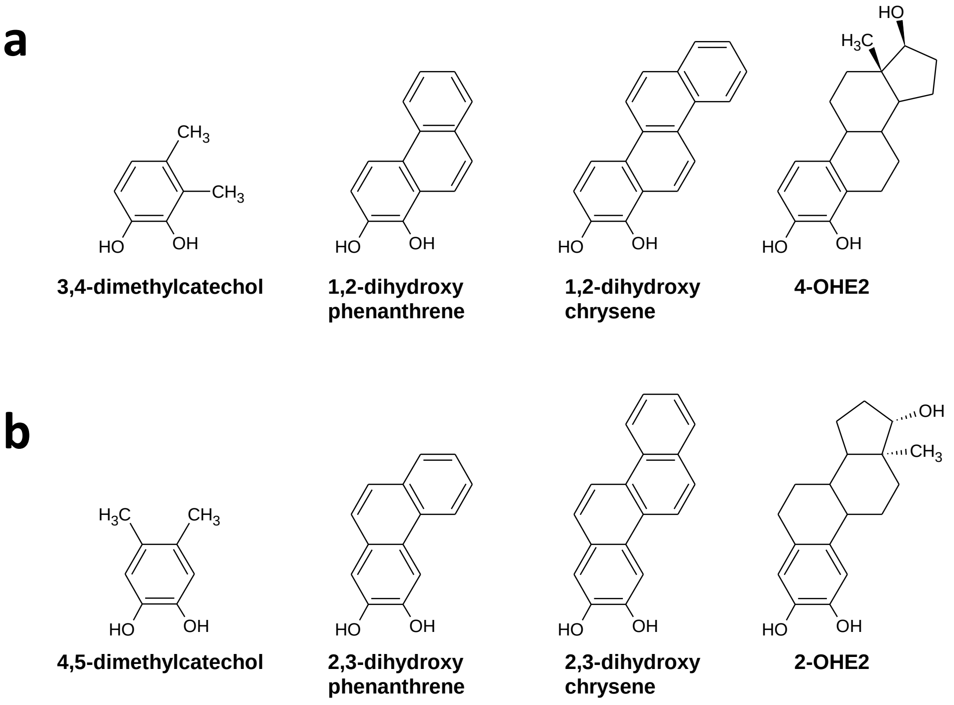

Supplementary Figure S1: **Dihydroxylated polycyclic aromatic hydrocarbons structures toward 4-OHE2 and 2-OHE2.** Structures of 3,4 (**a**) and 4,5 (**b**) substituted catechols were reported in comparison with 4-OHE2 (**a**) and 2-OHE2 (**b**) structures to highlight structure similarities.

a

ERCDs	Temperature of induction (°C)	Time of induction (h)	[IPTG] mM	[Fe(II)] µM	S.A. 2,3-DHBP mU/OD_{600}
**PP28735**	28	4	0.025	100	34.0±5.5
**PP26077**	28	2	0.4	100	196.0±15.7
**PP00124**	37	2	0.4	100	380.0±26.2
**PP00193**	28	4	0.025	100	988.0±18.2

b

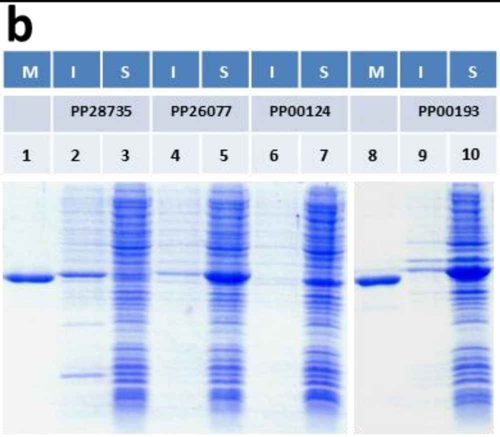

Supplementary Figure S2: ERCDs recombinant expression conditions. **a:** optimized recombinant expression conditions used for each protein and Specific Activity (S.A.) of ERCDs expressing cells (mU/OD_{600}) on 2,3-dihydroxybiphenyl (2,3-DHBP). **b:** SDS-PAGE analysis of ERCDs analytical expression experiments in *E. coli* BL21(DE3). M: 33 kDa marker. I: insoluble fractions of induced cultures. S: soluble fractions of induced cultures. The two original uncropped full length gels are reported in Supplementary Figure S5 A (left side gel, lanes from 1 to 7) and S5 B (right side gel, lanes from 8 to 10).

a

ERCDs	Initial total Units	Final total Units	Yield (%)
**PP28735**	18 $U_{2,3\text{-}DHBP}$	8 $U_{2,3\text{-}DHBP}$	44
**PP26077**	37 $U_{2,3\text{-}DHBP}$	30 $U_{2,3\text{-}DHBP}$	81
**PP00124**	88 $U_{2,3\text{-}DHBP}$	58 $U_{2,3\text{-}DHBP}$	66
**PP00193**	1476 $U_{3\text{-}MC}$	1406 $U_{3\text{-}MC}$	95

b

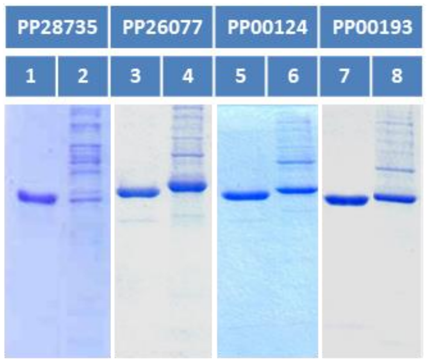

Supplementary Figure S3: **ERCDs purification. a:** total Units of ERCDs before and after the chromatographic step. Yield is reported as percentage of total units (errors are within 15%). **b:** analysis of the purified ERCDs. Lane 1-3-5-7: 33 kDa marker. Lane 2: purified PP28735 (lanes 1 and 2 were cropped from different parts of the same gel; the uncropped original gel is shown in Supplementary Figure S6 A). Lane 4: purified PP26077 (lanes 3 and 4 were cropped from adjacent parts of the same gel; the uncropped original gel is shown in Supplementary Figure S6 B). Lane 6: purified PP00124 (lanes 5 and 6 were cropped from adjacent parts of the same gel; the uncropped original gel is shown in Supplementary Figure S6 C). Lane 8: purified PP00193 (lanes 7 and 8 were cropped from different parts of the same gel; the uncropped original gel is shown in Supplementary Figure S6 D).

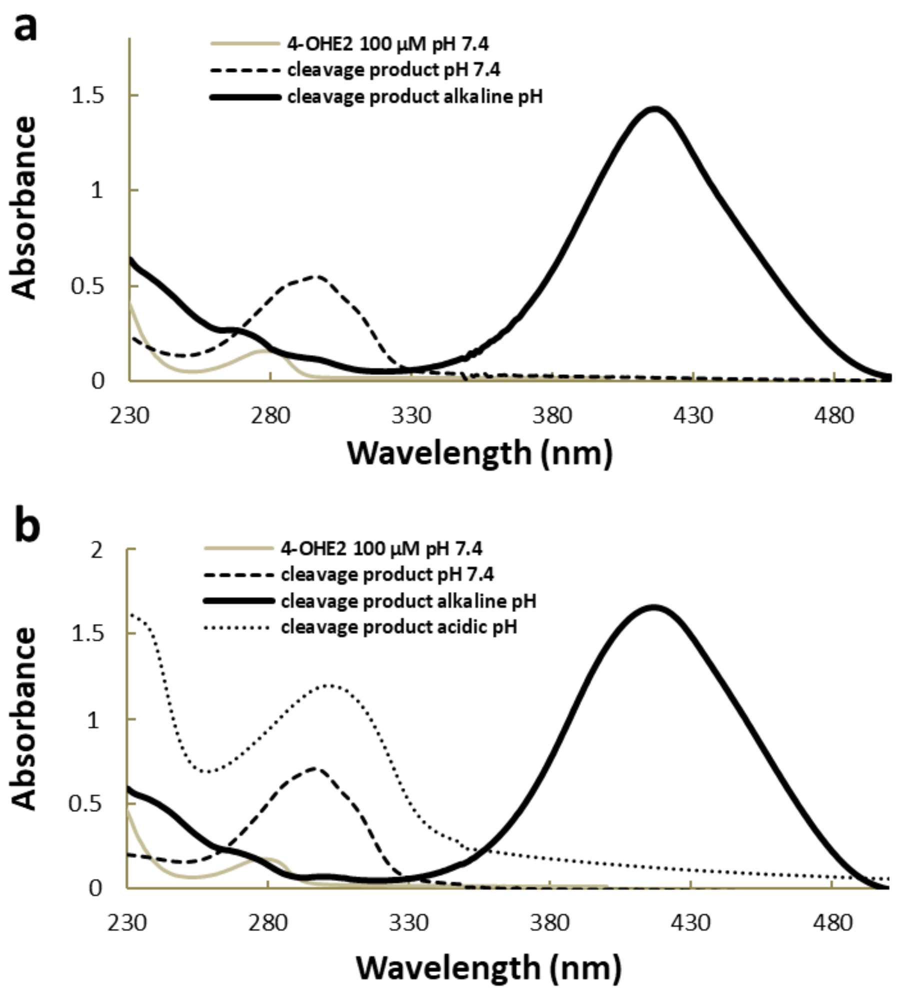

Supplementary Figure S4: UV-VIS spectral analyses of semialdehyde from 4-OHE2 as function of pH. Semialdehyde spectra were recorded at pH 7.5, acidic (1% formic acid) and alkaline (100 mM NaOH) pH. Reactions were carried out with PP28735 (**a**) and PP00124 (**b**) enzymes in 1 mL of 50 mM Tris/HCl pH 7.5 buffer containing 100 µM 4-OHE2 (gray lines, spectra at t=0). The reactions were started by adding 1 µg of purified enzymes. Semialdehyde production was monitored by the Scanning Kinetics program on Cary 100 UV-VIS spectrophotometer in a wavelength range from 230 to 500 nm, recording the spectra until 10 minutes, at 25°C, to obtain total conversion of 4-OHE2. At the end of cleavage reactions at pH 7.5 (dashed lines, spectra at the end of reactions), NaOH (100 mM final concentration) was added to alkalinize reactions and convert the semialdehyde into di-anionic yellow form (solid black bold lines). Furthermore, another sample was added with formic acid (1% final concentration) to analyze spectral properties useful for HPLC analyses (dotted line).

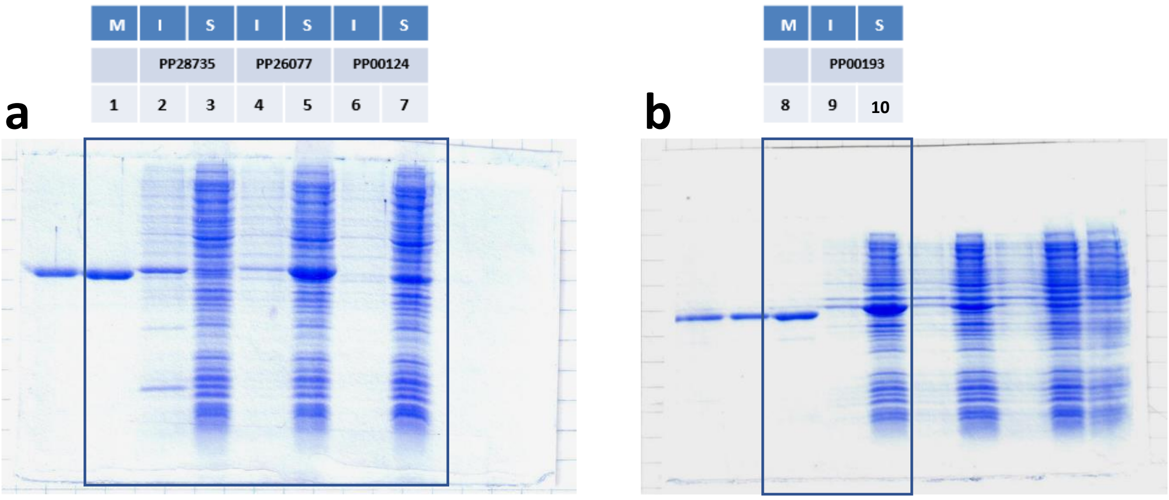

Supplementary Figure S5: **ERCDs recombinant expression.** The original uncropped full length images of SDS-PAGEs reported in Supplementary Figure S2 B are shown. Gels were dried on paper after Coomassie brilliant blue G-250 staining. The boxed lanes correspond to (**a**) lanes 1-7 of the left side gel and (**b**) lanes 8-10 of the right side gel showed in Supplementary Figure S2 B.

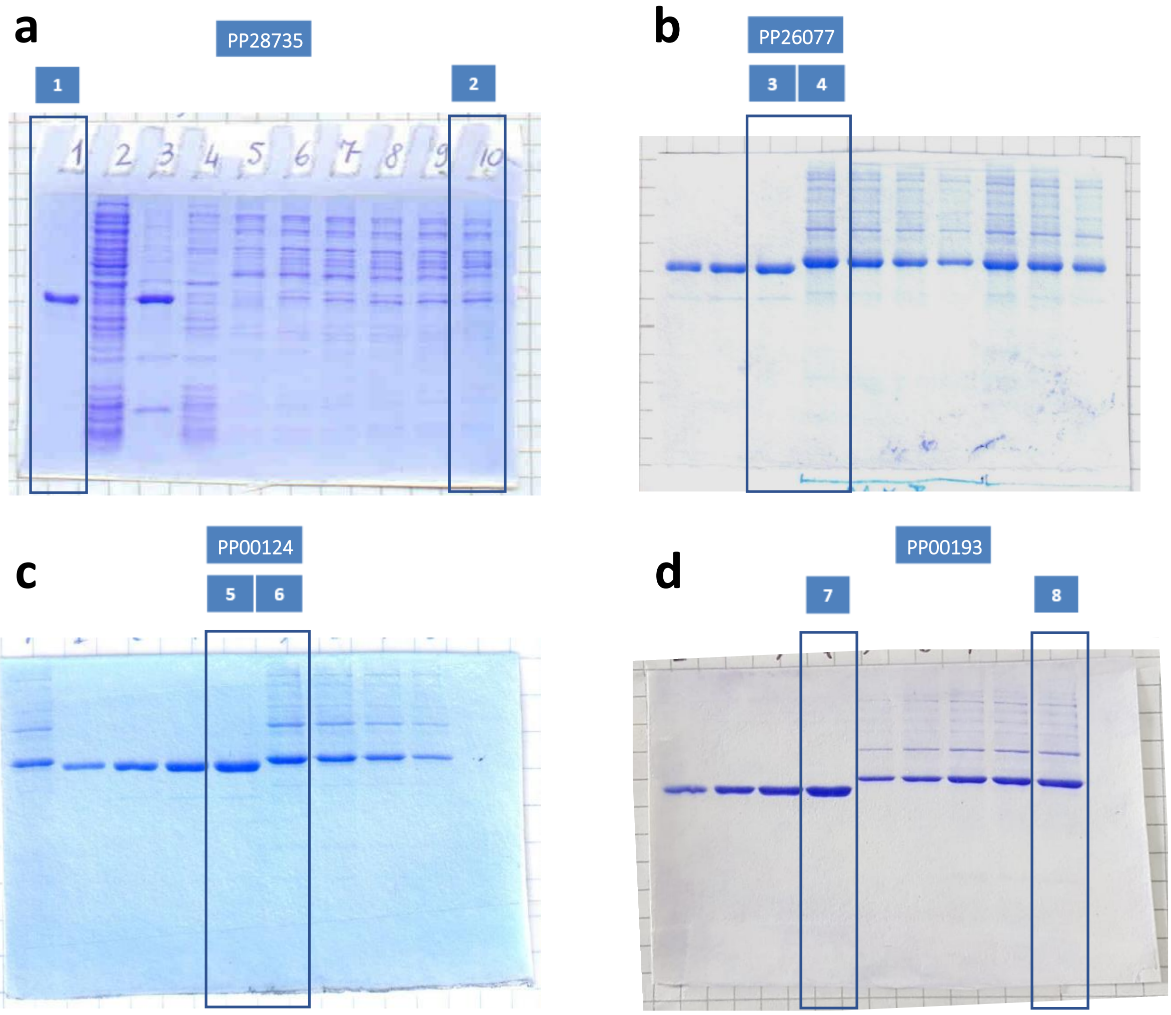

Supplementary Figure S6: **SDS-PAGE analyses of purified ERCDs.** The original uncropped full length images of SDS-PAGEs reported in Supplementary Figure S3 B are shown. Gels were dried on paper after Coomassie brilliant blue G-250 staining. The boxed lanes correspond to lanes 1-2 (**a**), 3-4 (**b**), 5-6 (**c**) and 7-8 (**d**) of the gels showed in Supplementary Figure S3 B.

Supplementary Tables.

ERCDs	expression levels (mg/L)	Protein with Fe(II) (%)	Protein with Fe(III) (%)	Protein without iron (%)
**PP26077**	120	60	15	25
**PP00124**	40	100	0	0
**PP00193**	100	88	5	7

Supplementary Table S1: **Quantification of total iron amount in *N*ovosphingobium sp. PP1Y ERCDs**.

**Substrates**			**Products (cis-muconic semialdehydes - IUPAC)**		
	λ_{max}	ε (acidic pH) mM^{-1} cm^{-1}		λ_{max}	ε (pH 7.5) mM^{-1} cm^{-1}
**catechol**	275	2.34	**(2E,4Z) 2-hydroxy-6-oxohexa-2,4-dienoic acid**	375	33
**3 – methylcatechol (3MC)**	278	1.48	**(2E,4Z) 2-hydroxy-6-oxohepta-2,4-dienoic acid**	388	13.8
**4 – methylcatechol (4MC)**	280	2.45	**(2E,4Z) 2-hydroxy-5-methyl-6-oxohexa-2,4-dienoic acid**	382	28.1
**2,3 – dihydroxybiphenyl (2,3-DHBP)**	282	1.48	**(2E,4Z) 2-hydroxy-5-methyl-6-oxohepta-2,4-dienoic acid**	434	13.2

Supplementary Table S2: Catecholic substrates and semialdehydes products absorbance maximum wavelength (λ_{max}) and extinction coefficients (ε, $mM^{-1}cm^{-1}$). Substrates: These parameters were used to calculate stock solutions concentrations. **Products**: These parameters were used to calculate semialdehydes concentration at neutral pH, in the enzymatic assay conditions. Semialdehydes IUPAC names are indicated.
